# The regulation of oxidative phosphorylation pathway on *Vibrio alginolyticus* adhesion under adversities

**DOI:** 10.1002/mbo3.805

**Published:** 2019-02-14

**Authors:** Li Huang, Lixing Huang, Lingmin Zhao, Yingxue Qin, Yongquan Su, Qingpi Yan

**Affiliations:** ^1^ State Key Laboratory of Large Yellow Croaker Breeding Ningde Fujian China; ^2^ Key Laboratory of Healthy Mariculture for the East China Sea, Ministry of Agriculture Fisheries College, Jimei University Xiamen Fujian China

**Keywords:** adhesion, environmental stresses, oxidative phosphorylation pathway, *Vibrio alginolyticus*

## Abstract

*Vibrio alginolyticus* is one of the most important pathogens in mariculture and leading to heavy losses. After treatment with Cu^2+^, Pb^2+^, and low pH, the expression of oxidative phosphorylation pathway genes, including *coxA*, *coxB*, *coxC*, *ccoN*, *ccoO*, and *ccoQ*, was found commonly downregulated by RNA‐seq as well as quantitative real‐time PCR. RNAi significantly reduced the expression of *coxA*, *coxB*, *coxC*, c*coN*, *ccoO*, and *ccoQ* in *V. alginolyticus*. Compared with the wild‐type strain, the adhesion abilities of RNAi strains of *V. alginolyticus *were significantly impaired, as well as their cytochrome C oxidase activity. *ccoQ *appeared to be more important in the regulation of bacterial adhesion in these target genes, while *ccoO* was relatively weak in the regulation of the adhesion. Meanwhile, the changes of temperature, salinity, pH, and starvation affected *coxA*, *coxB*, *coxC*, *ccoN*, *ccoO*, and *ccoQ* expression remarkably. These findings indicated that: the oxidative phosphorylation pathway is a critical regulator of adhesion in *V. alginolyticus*; *coxA*, *coxB*, *coxC*, *ccoN*, *ccoO*, and *ccoQ* regulate the bacterial adhesion in response to environmental changes such as temperature, salinity, pH, and starvation.

## 1 INTRODUCTION

Many biological processes depended on the adenosine triphosphate (ATP, the universal cellular energy currency) released (Llorente‐Garcia et al., 2014). Adenosine triphosphate can be gained by oxidative phosphorylation and by substrate level phosphorylation (SLP), or more generally electron transport phosphorylation (ETP) in many aerobic respiring bacteria (Koch‐Koerfges, Kabus, Ochrombel, Marin, & Bott, 2012). For bacteria, the oxidative phosphorylation is a major metabolic pathway to obtain energy required for cell growth and reproduction. Five major protein complexes (complexes I–IV) which constitute the electron transport chain drive the oxidative phosphorylation (Koo et al., 2015; Mitchell, 1961, 2011). The oxidative phosphorylation was demonstrated to influence the survival ability of *Escherichia coli *in stationary phase at alkaline pH (Weiner & Model, 1994). Recently, many researches revealed the major roles of temperature, salinity, pH and starvation in oxidative phosphorylation. Jarmuszkiewicz, Woyda‐Ploszczyca, Koziel, Majerczak, and Zoladz (2015) also found that the efficiency of oxidative phosphorylation was decreased with the temperature increasing from 25 to 42°C. Xu et al. (2015) and Hu, Kang, Tang, and Lee (2015) indicated that the oxidative phosphorylation could be affected by salinity. The pH could significantly change the activity of oxidative phosphorylation (Korzeniewski, 2015). As Monternier et al. (2015) described, the activity of oxidative phosphorylation was sensitive to starvation.

*Vibrio alginolyticus*, a moderately halophilic gram‐negative bacillus, is considered to be an important opportunistic pathogens in mariculture Luo et al. (2016). *Vibrio alginolyticus* was associated with epidemics of maricultured animals, such as coral reefs (Xie et al., 2013), shellfish (Mechri, Monastiri, Medhioub, Medhioub, & Aouni, 2017), fish (Cao et al., 2018), and crustaceans (Gomez‐Gil et al., 1998), and resulted in heavy economy losses. It has also been reported to cause disease in human beings during warm periods which may lead to otitis and wound infections (Campanelli, Sanchez‐Politta, & Saurat, 2008). The adhesion of pathogen to the surfaces of host is one of the key steps in the initial stage of infection (Chen, Yan, Ma, Zhuang, & Wang, 2007), and many pathogenic bacteria have been found to have strong adhesion ability to their hosts (Qin, Lin, Chen, Xu, & Yan, 2016; Lin et al., 2017), and the bacterial adhesion was regulated by different genes (Huang, Qin et al., 2015). Environmental conditions were proved to be capable of affecting the adhesion ability of bacteria (Yan, Chen, Ma, Zhuang, & Wang, 2007). The temperature, salinity, pH, and starvation play the important roles of environmental conditions in the process of bacterium to adhere to fish mucus (Yan, Zhao, Wang, Zou, & Chen, 2010).

Although there are a few literatures about the molecular mechanism of *V. alginolyticus* adhesion in recent years, there are few literatures about the molecular mechanism of *V. alginolyticus* adhesion regulation under diverse environments. Furthermore, no study has been reported about the relationship between oxidative phosphorylation pathway and the adhesion of pathogenic bacteria. For further understanding of the *V. alginolyticus* adhesion regulation, the RNA‐seq was carried out on *V. alginolyticus *which received the treatments of low pH, Pb^2+^, and Cu^2+^ (Kong et al., 2015).

In the present study, the bioinformatics analysis about the oxidative phosphorylation pathway was carried out, following by quantitative real‐time PCR (qRT‐PCR), RNA interference (RNAi), and in vitro adhesion assay. The purpose of this study is to reveal the relationship between bacterial adhesion and oxidative phosphorylation pathway under adversities.

## 2 MATERIALS AND METHODS

### 2.1 Bacterial strains, culture, and media

*Vibrio alginolyticus* high pathogenic strain ND‐01 was isolated from mariculture fish large yellow croaker (Yan, Wang, & Su 2001). *Vibrio alginolyticus* was cultured on LB agar or Luria–Bertani (LB) broth added with 2% NaCl at 28°C (Huang, Xu, Su, Zhao, & Yan, 2018).

For further validation of the RNA‐seq, treatments with low pH (pH 5), Pb (100 mg/L), and Cu (50 mg/L) were carried out on *V. alginolyticus*. The control group was cultured in LB broth (added with 2% NaCl, pH = 7) (Guo et al., 2018). There were six replicates for each treatment. After overnight culture at 28°C, the bacterial cell were collected and used for RNA extraction.

In order to explore the influence of temperature, *V. alginolyticus* was grown at 4, 15, 28, 37, and 44°C, respectively, in LB broth (Huang et al., 2017).

To evaluate the influence of various pH values, *V. alginolyticus* was grown at pH 5, 6, 7, 8, and 9, respectively, and then washed with PBS (pH = 5, 6, 7, 8, and 9, respectively) (Huang, Huang et al., 2016).

For evaluation of the effect of salinity, *V. alginolyticus* was grown in LB broth with salinities as 0.8%, 1.5%, 2.5%, 3.5%, and 4.5%, and then washed with PBS with corresponding salinities and adjusted to OD_560_ ≈ 0.3.

For evaluation of the adhesion of starved cells, *V. alginolyticus* was adjusted to OD_560 _≈ 0.3 in normal PBS and kept starvation at 28°C for 1, 3, 5, and 7 days, respectively (Yi et al., 2008).

### 2.2 Functional classification and enrichment analysis for differential expression genes

As our previously reported, the Blast2GO was applied to get Gene Ontology (GO) annotation of the unigenes, while WEGO was applied for GO functional classification analysis of all genes and understand the distribution of gene functions at the macro level. GO terms with Bonferroni corrected *p* value ≤ 0.05 were identified as significantly enriched in differential expression genes (DEGs) (Sun, Leo et al., 2018; Zhang et al., 2018).

Software of Blastall against KEGG database (http://www.genome.jp/kegg/) was used for the KEGG pathways annotation. *Q* value ≤ 0.05 was taken as a threshold, while pathways fulfilling this were regarded as significantly enriched pathways in DEGs (Huang, Liu et al., 2018).

### 2.3 Preparation of mucus

Healthy marine cultured large yellow croakers from Ningde (Fujian, China) were used for mucus collection via our previously described procedure (Huang, Huang et al., 2016). The skin was first washed with sterile PBS (0.01 mol/L, pH 7.2). Then, the skin mucus was harvested by scraping off the surface of the skin. The mucus was then homogenized in PBS, removed particulate materials by centrifugation twice (20,000 g, 4°C, 30 min), filtered through 0.45‐ and 0.22‐μm pore size filters successively, and finally, adjusted in PBS to 1 mg protein/ml.

### 2.4 Total RNA extraction and reverse transcription

Trizol (Invitrogen) was used to extract the total RNA from the bacterial cells following the manufacturer's protocol. A Rever Aid Mu‐MLV cDNA synthesis kit (TransGen Biotech) was used to synthesize first‐strand complementary (c) DNA from 2.0 mg total RNA following the manufacturer's protocol (Huang, Zuo, & Jiang, 2019).

### 2.5 Transient gene silencing

According to the gene sequence, short interfering RNA (siRNA) (21–23 nt) with a characteristic and highly specific 2‐ to 3‐nucleotide 3′ overhang was obtained from GenePharma Co. Ltd. Sequences of siRNA used here are listed in Table 1.

**Table 1:** siRNA sequences

Target gene	siRNA sequences
*ccoN*	F: 5′ GCUCUGUUCGCAACGUCUUTT 3′
	R: 5′ AAGACGUUGCGAACAGAGCTT 3′
*ccoO*	F: 5′ GCUCAGAGACUGAACGUUATT 3′
	R:5′ UAACGUUCAGUCUCUGAGCTT 3′
*ccoQ*	F: 5′ GCAUCGUAUGGUGGGCAUUTT 3′
	R: 5′ AAUGCCCACCAUACGAUGCTT 3′
*coxA*	F: 5′ GCAUUUACGGGCCUUGCUATT 3′
	R: 5′ UAGCAAGGCCCGUAAAUGCTT3′
*coxB*	F: 5′ CCAUCUUUCGUCAUCGCAATT 3′
	R: 5′ UUGCGAUGACGAAAGAUGGTT3′
*coxC*	F: 5′ GGAUGAGCUGGUUCAUCUUTT 3′
	R: 5′ AAGAUGAACCAGCUCAUCCTT 3′
Negative control	F: 5′ UUCUCCGAACGUGUCACGUTT 3′
	R: 5′ ACGUGACACGUUCGGAGAATT 3′

Note

siRNA: short interfering RNA.

A mixture of 5 μl 20 μmol/L siRNA and 100 μl *V. alginolyticus* competent cells was transferred to the cuvette after being chilled on ice for 30 min, followed by electroporation (1.8 kV, 6 ms) with a Bio‐Rad MicroPulser (Bio‐Rad Laboratories, Inc.). After adding 900 μl of LB immediately, the mixture was incubated at 28°C for 1, 3, 6, 9, and 12 hr prior to RNA extraction, qRT‐PCR, and in vitro adhesion assay (Huang, Hu et al., 2016).

### 2.6 Stable gene silencing

*coxA*, *coxB*, *coxC*, *ccoN*, *ccoO*, and *ccoQ* were stable silenced by vectors containing short hairpin RNA (shRNA) sequences targeting their coding regions. The shRNAs were obtained from Shanghai Generay Biotech Co., Ltd. (Table 2). After ligating annealed oligonucleotides into the Tc operon of *BamHI* and *SphI* double digested pACYC184 vector with T4 DNA ligase (Qin et al., 2014), heat shock was introduced to transform recombinant plasmids into *E. coli* SM10. The recombinant plasmids were then transferred from *E. coli* SM10 into *V. alginolyticus* by conjugation. The empty pACYC184 vector was taken as the control. Chloramphenicol (34 μg/ml) was used to screen stable silenced clones (Huang, Xu et al., 2018).

**Table 2:** Oligo nucleotides used in producing shRNA for stable gene silencing

Target gene	siRNA sequences (5′–3′)
*ccoN*	F: GATCCGCTCTGTTCGCAACGTCTTTTTTCAAGAGAAAAAGACGTTGCGAACAGAGCTTTTTTGCATG
	R: CAAAAAAGCTCTGTTCGCAACGTCTTTTTCTCTTGAAAAAAGACGTTGCGAACAGAGCG
*ccoO*	F: GATCCGCTCAGAGACTGAACGTTATTTTCAAGAGAAATAACGTTCAGTCTCTGAGCTTTTTTGCATG
	R: CAAAAAAGCTCAGAGACTGAACGTTATTTCTCTTGAAAATAACGTTCAGTCTCTGAGCG
*ccoQ*	F: GATCCGCATCGTATGGTGGGCATTTTTTCAAGAGAAAAATGCCCACCATACGATGCTTTTTTGCATG
	R:CAAAAAAGCATCGTATGGTGGGCATTTTTCTCTTGAAAAAATGCCCACCATACGATGCG
*coxA*	F: GATCCGCATTTACGGGCCTTGCTATTTTCAAGAGAAATAGCAAGGCCCGTAAATGCTTTTTTGCATG
	R: CAAAAAAGCATTTACGGGCCTTGCTATTTCTCTTGAAAATAGCAAGGCCCGTAAATGCG
*coxB*	F: GATCCCCATCTTTCGTCATCGCAATTTTCAAGAGAAATTGCGATGACGAAAGATGGTTTTTTGCATG
	R: CAAAAAACCATCTTTCGTCATCGCAATTTCTCTTGAAAATTGCGATGACGAAAGATGGG
*coxC*	F: GATCCGGATGAGCTGGTTCATCTTTTTTCAAGAGAAAAAGATGAACCAGCTCATCCTTTTTTGCATG
	R: CAAAAAAGGATGAGCTGGTTCATCTTTTTCTCTTGAAAAAAGATGAACCAGCTCATCCG

Note

shRNA: short hairpin RNA; siRNA: short interfering RNA.

### 2.7 qRT‐PCR assay

A QuantStudio™ 6 Flex real‐time PCR system (ABI) was used to carry out qRT‐PCR with the SYBR green I fluorescent dye (Dongsheng) in accordance with the manufacturer's instructions (Liu et al., 2017). The expression levels of *coxA*, *coxB*, *coxC*, *ccoN*, *ccoO*, and *ccoQ* were normalized to that of 16S RNA (*n* = 6). Relative Expression Software Tool (REST 2008‐version 2) was used to assess the relative expression levels of *coxA*, *coxB*, *coxC*, *ccoN*, *ccoO*, and *ccoQ* (Pfaffl, Horgan, & Dempfle, 2002). Primers used here are listed in Table 3.

**Table 3:** Primers used in quantitative real‐time PCR

Primer	Sequence
*ccoN*‐for	5′ ACGCACTTTCTCACTACAC 3′
*ccoN*‐rev	5′ GACGCTTCTACAGACTCAAC 3′
*ccoO*‐for	5′ GTGACATCTACATCCGTGAAG 3′
*ccoO*‐rev	5′ CTTCTGAGTTAGCTTACCGTC 3′
*ccoQ*‐for	5′ CGGTACAATTCATAGTATTTATAC 3′
*ccoQ*‐rev	5′ CTTCACTCCTTGGTTATTTG 3′
*coxA*‐for	5′ CAATATGTACGACCACAAGC 3′
*coxA*‐rev	5′ TGGCACTGTCCATTCTAAG 3′
*coxB*‐for	5′ CGTCATCGCAAGTCAAAAGGAG 3′
*coxB*‐rev	5′ GGCTGGTGGCAAGCAAACTG 3′
*coxC*‐for	5′ CGCCAGGATGATTTCTGTGCC 3′
*coxC*‐rev	5′ GGAAAAAGCCCGCAAGCACG 3′
16S‐for	5′ GGGGAGTACGGTCGCAAGAT 3′
16S‐rev	5′ CGCTGGCAAACAAGGATAAGG 3′

### 2.8 Determination of cytochrome C oxidase activity

The procedure for determining the cytochrome C oxidase (CoxC) activity of *V. alginolyticus *was performed according to the instructions of Sigma–Aldrich Cytochrome C Oxidase Activity Assay kit. Prior to starting the assay, the multifunctional microplate reader was incubated at room temperature and set the absorbance of 550 nm. Then, the multifunctional microplate reader was used to read data once every 10 s interval within 1 min. Finally, the activity of CoxC of each experimental group was calculated according to the formula in the instructions.

### 2.9 In vitro adhesion assay

According to the procedure described by Kong et al. (2015), in vitro adhesion assay was carried out. A quantity of 50 μl of mucus was evenly spread on a glass slide (22 × 22 mm) and fixed by methanol for 20 min. Then, 1 ml of bacterial suspension (10^8 ^CFU/ml) was placed on the mucus‐coated glass slides, following by incubating humid at 28°C for 2 hr, and washing five times with PBS. The adherent bacteria were then fixed for 30 min by 4% methanol, dyed for 3 min by crystal violet, and counted with a microscope (×1,000). Each group was conducted for five times, and 20 fields of view were selected randomly. Negative control was performed using PBS instead of bacterial suspension.

### 2.10 Data processing

Results were showed as mean ± *SD* and statistically analyzed with spss 18.0. Differences were determined by analysis of one‐way ANOVA followed by Dunnett's multiple comparison tests. A value of *p* < 0.05 was considered statistically significant.

## 3 RESULTS

### 3.1 DEGs screening by RNA‐seq

Results of previous study (Kong et al., 2015) showed that the low pH, Pb^2+, ^and Cu^2+^ could significantly reduce the adhesion ability of *V. alginolyticus* by 56.58%, 39.26%, and 37.41%, respectively. Therefore, RNA‐seq was performed on *V. alginolyticus *which was treated with low pH, Pb^2+ ^and Cu^2+^.

Differential expression genes analysis yielded 1,791, 1,085, and 1,637 DEGs in the low pH‐, Pb^2+^‐ and Cu^2+^‐treated groups and compared with the control group. Then, GO and KEGG analysis were also performed according to the previously described by Huang, Liu et al. (2018) in this research, which showed that the oxidative phosphorylation pathway was worthy for further research to validate its role in adhesion regulation.

**Figure 1:**
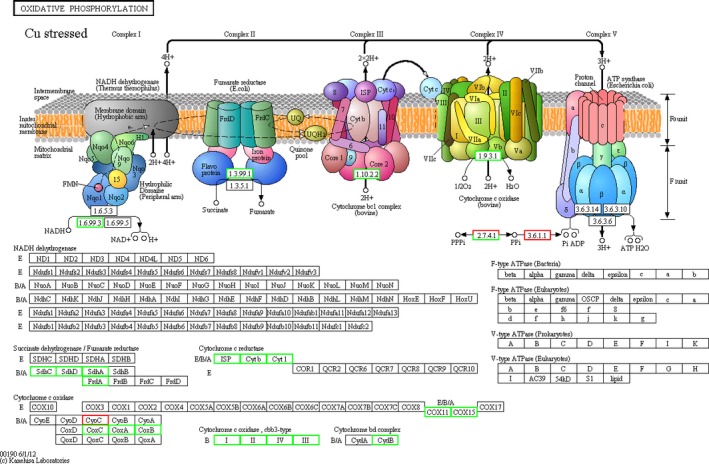
The overall scheme for genes involved in the oxidative phosphorylation pathway which were significantly affected by Cu stress. Green and red indicated down‐ and upregulation, respectively

**Figure 2:**
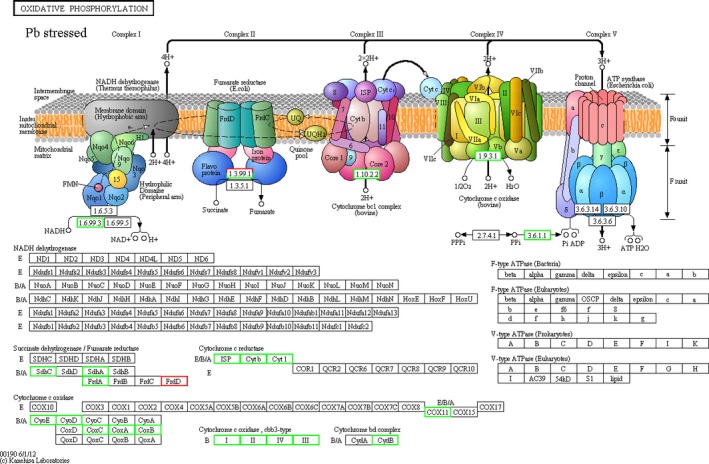
The overall scheme for genes involved in the oxidative phosphorylation pathway which were significantly affected by Pb stress. Green and red indicated down‐ and upregulation, respectively

**Figure 3:**
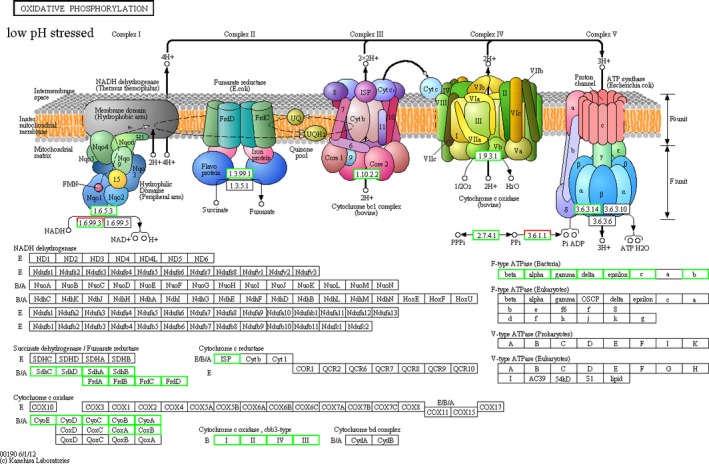
The overall scheme for genes involved in the oxidative phosphorylation pathway which were significantly affected by low pH stress. Green and red indicated down‐ and upregulation, respectively

There were six commonly downregulated DEGs in the oxidative phosphorylation pathway, and these DEGs were significantly changed in the Cu^2+^, Pb^2+, ^and low pH groups (Figure1, 2, 3). Therefore, they appeared to be associated with the change of environmental factors. These six DEGs were subunit I of aa_3_‐type cytochrome c oxidase gene (*coxA*), subunit II of aa_3_‐type cytochrome c oxidase gene (*coxB*), subunit III of aa_3_‐type cytochrome c oxidase gene (*coxC*), subunit I of cbb_3_‐type cytochrome oxidase gene (*ccoN*), subunit II of cbb_3_‐type cytochrome oxidase gene (*ccoO*), and subunit IV of cbb_3_‐type cytochrome oxidase gene (*ccoQ*), respectively.

### 3.2 Validation of the results of RNA‐seq

**Figure 4:**
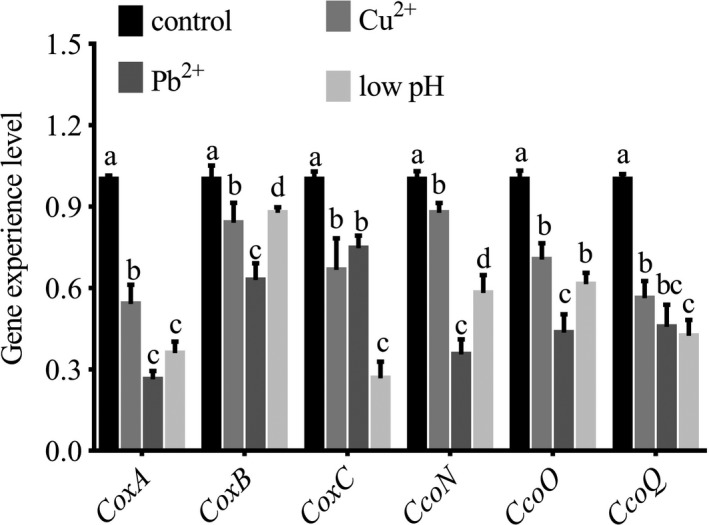
Expression level of *coxA*, *coxB*, *coxC*, *ccoN*, *ccoO*, and *ccoQ *after stress treatments detected by quantitative real‐time PCR. Data are presented as the means ± *SD*, *n* = 6. Means of the treatments not sharing a common letter are significantly different at *p* < 0.05

Quantitative real‐time PCR on *coxA*, *coxB*, *coxC*, *ccoN*, *ccoO*, and *ccoQ* was carried out to validate the results of RNA‐seq. The results of qRT‐PCR matched those of the RNA‐seq: the low pH, Pb, and Cu treatments significantly reduced the expression of *coxA* (by 2.78‐, 3.80‐, and 1.85‐fold, respectively), *coxB* (by 1.14‐, 1.59‐, and 1.19‐fold, respectively), *coxC* (by 3.75‐, 1.34‐, and 1.50‐fold, respectively), *ccoN* (by 1.72‐, 2.82‐, and 1.14‐fold, respectively), *ccoO *(by 1.63‐, 2.30‐, and 1.42‐fold, respectively), and *ccoQ *(by 2.36‐, 2.19‐, and 1.78‐fold, respectively) (Figure 4). These reinforced the reliability of the sequencing data.

### 3.3 Influence of transient gene silencing

**Figure 5:**
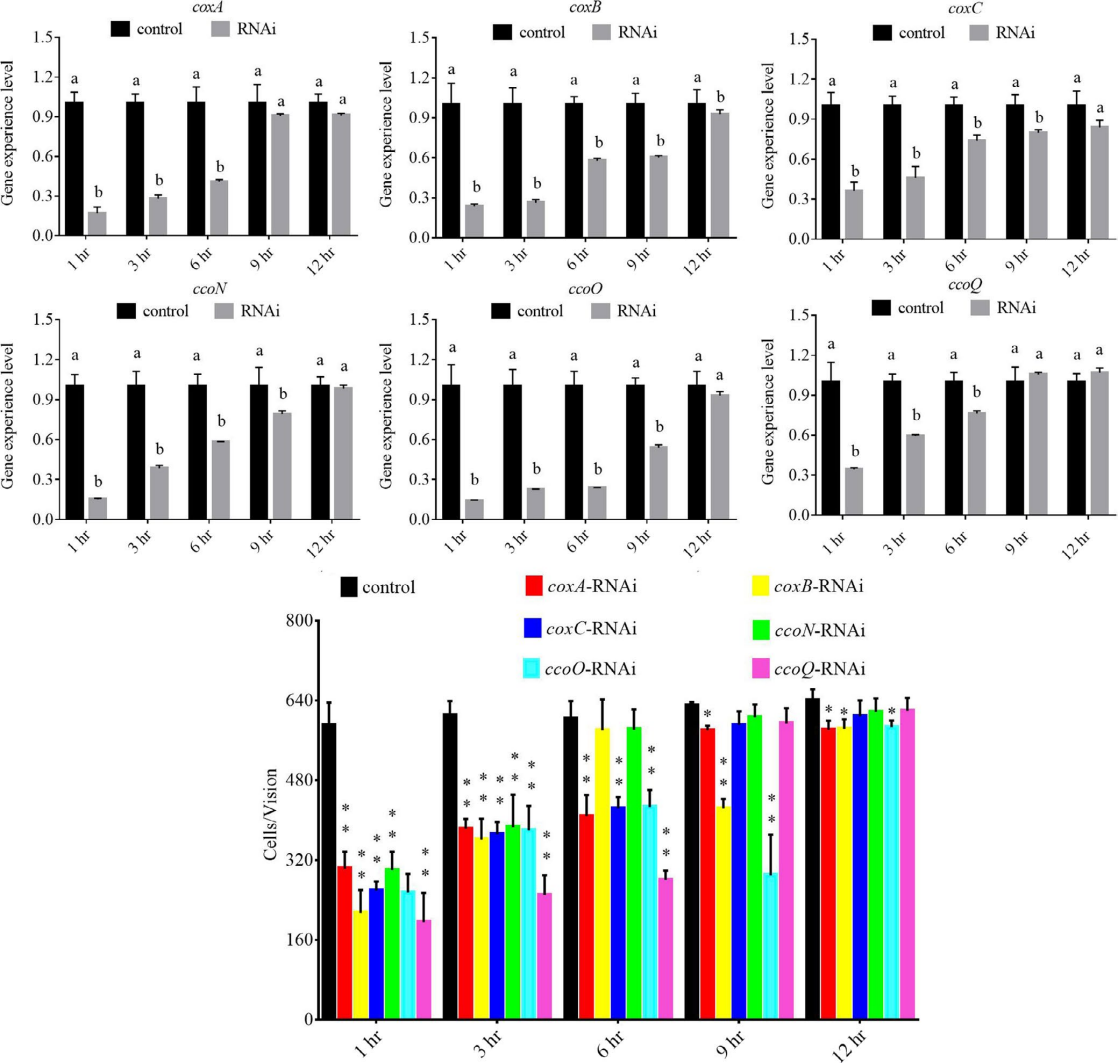
Transient RNAi reduced the adhesion of *Vibrio alginolyticus. *(a) Quantitative real‐time PCR analysis of the expression of *coxA*, *coxB*, *coxC*, *ccoN*, *ccoO*, and *ccoQ *after transient gene silencing at 1, 3, 6, 9, and 12 hr. Data are presented as the means ± *SD*, *n* = 6. Means of the treatments not sharing a common letter are significantly different at *p* < 0.05. (b) The adhesion capacity to mucus of transient silenced *V. alginolyticus* at 1, 3, 6, 9, and 12 hr. Data are presented as the means ± *SD*, *n = *3. **p < 0.01 versus the control group. *p < 0.05 versus the control group.

After being treated with siRNAs, the gene expression levels of *V. alginolyticus *were detected at 1, 3, 6, 9, and 12 hr. The expression levels of these target genes were normalized against the control (scrambled) siRNA treatments. The expression of these genes decreased significantly at 1–6 hr. After 6 hr, *coxA* and *ccoQ* were no longer changed significantly, while *coxB*, *coxC*, *ccoN*, and *ccoO* were remain significantly changed at 12 hr (Figure 5a). The decreased expression of these genes indicated proper work of these siRNAs.

Our previously study results indicated that the siRNA was up‐taken into *V. alginolyticus* at a relatively high efficiency (Huang, Hu et al., 2015). The *V. alginolyticus* adhesion ability with and without RNAi treatments were compared. In vitro adhesion assay showed that RNAi significantly reduced *V. alginolyticus* adhesion (Figure 5b), while the downregulation of adhesion was alleviated as time went by. After RNAi, the trend of qRT‐PCR and in vitro adhesion assay results were quite similar. These results showed that RNAi of these genes could significantly impair the *V. alginolyticus* adhesion ability.

According to the results of data analysis, siRNA treatment significantly reduced *coxA *(by 5.85‐fold), *coxB *(by 4.20‐fold), *coxC *(by 2.77‐fold), *ccoN *(by 6.58‐fold), *ccoO *(by 7.08‐fold), and *ccoQ *(by 2.91‐fold) at 1 hr. Simultaneously, the adhesion of *V. alginolyticus *was significantly reduced by 1.97‐, 2.76‐, 2.28‐, 1.97‐, 2.32‐, and 3.02‐fold, respectively. Thus, *ccoQ *appeared to be more important in the regulation of bacterial adhesion in these target genes, while *ccoO *gene was relatively weak in the regulation of the adhesion.

### 3.4 Influence of stable gene silencing

#### 3.4.1 qRT‐PCR results of DEGs in the oxidative phosphorylation pathway after stable gene silencing

**Figure 6:**
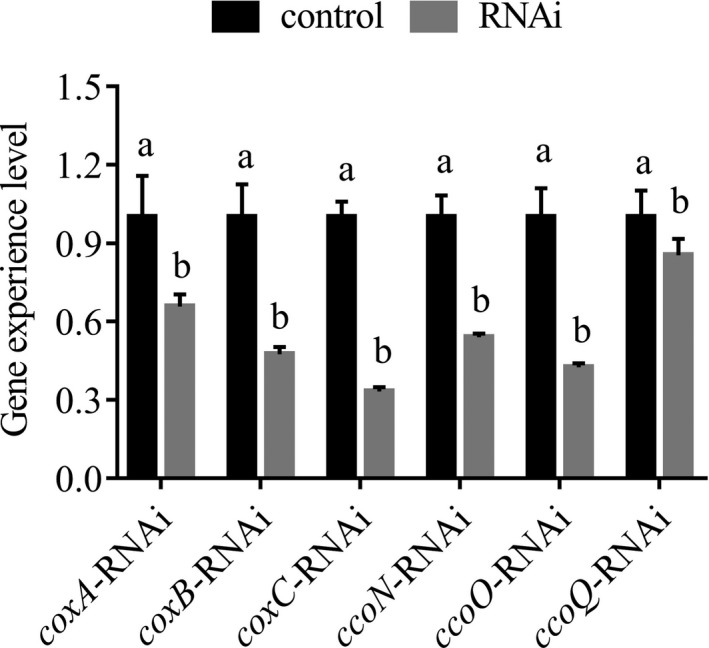
Quantitative real‐time PCR results of *coxA*, *coxB*, *coxC*, *ccoN*, *ccoO*, and *ccoQ* in the oxidative phosphorylation pathway after stable gene silencing. Data are presented as the means ± *SD*, *n* = 6. Means of the treatments not sharing a common letter are significantly different at *p* < 0.05

The results in Figure 6 showed that the expression levels of *coxA*, *coxB*, *coxC*, *ccoN*, *ccoO*, and *ccoQ *were decreased significantly in stable silenced strains by 1.52‐, 2.10‐, 3.00‐, 1.85‐, 2.35‐, and 1.17‐fold, respectively. The expression level of *coxC *gene was the most impaired among six genes in the oxidative phosphorylation pathway.

#### 3.4.2 Adhesion assay of *V. alginolyticus* after stable gene silencing

**Figure 7:**
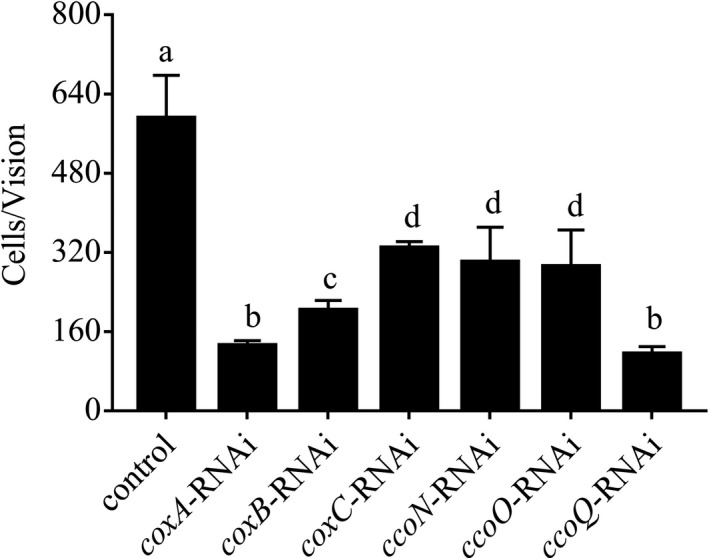
Stable RNAi reduced the adhesion of *Vibrio alginolyticus*. Data are presented as the means ± *SD*, *n* = 6. Means of the treatments not sharing a common letter are significantly different at *p* < 0.05

The adhesion ability of the control *V. alginolyticus *and stable silenced clones were shown in Figure 7. For the control group, 563 cells/view adhered to the slides, while the adhesion ability of *coxA*, *coxB*, *coxC*, *ccoN*, *ccoO*, and *ccoQ*‐RNAi *V. alginolyticus* were impaired by 77.80%， 65.72%， 44.40%， 49.20%， 50.80%, and 80.64%, respectively. The decrease of the adhesion ability of *ccoQ*‐RNAi *V. alginolyticus* was the largest among them, while the decrease of the adhesion ability of *coxC*‐RNAi *V. alginolyticus* was the least. These results indicated that stable gene silencing could significantly reduce the adhesion of *V. alginolyticus*.

#### 3.4.3 Enzyme activity of cytochrome C oxidase of *Vibrio alginolyticus* after stable gene silencing

**Figure 8:**
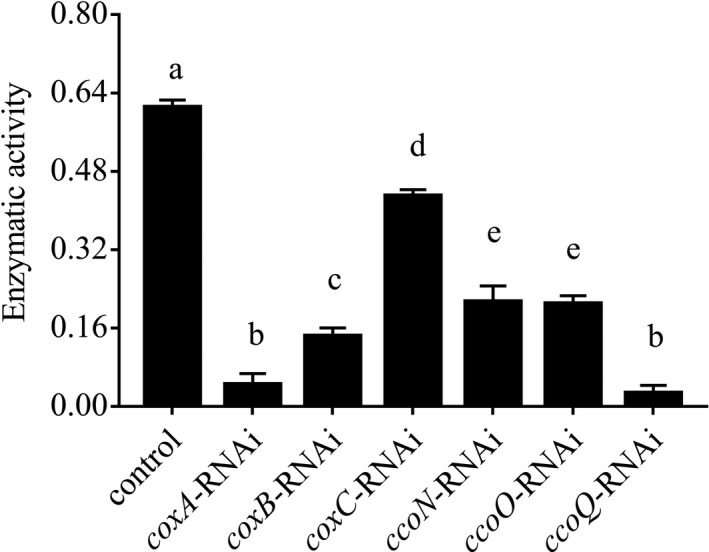
Stable gene silencing reduced the enzyme activity of cytochrome C oxidase of *Vibrio alginolyticus*. Data are presented as the means ± *SD*, *n* = 6. Means of the treatments not sharing a common letter are significantly different at *p* < 0.05

The results in Figure 8 showed that the control *V. alginolyticus* displayed well enzyme activity of CoxC and the stable silenced clones displayed defective enzyme activity of CoxC. The enzyme activity of CoxC of *coxA*, *coxB*, *coxC*, *ccoN*, *ccoO*, and *ccoQ*‐RNAi *V. alginolyticus* were impaired by 92.78%, 76.63%, 29.73%, 65.12%, 65.81%, and 95.70%, respectively. The enzyme activity of CoxC of *coxA‐ *and *ccoQ*‐RNAi *V. alginolyticus* were more impaired among them, and the *coxC*‐RNAi *V. alginolyticus *was less impaired. These results indicated that stable gene silencing of these six genes could significantly reduce the enzyme activity of CoxC.

### 3.5 Influence of temperature

**Figure 9:**
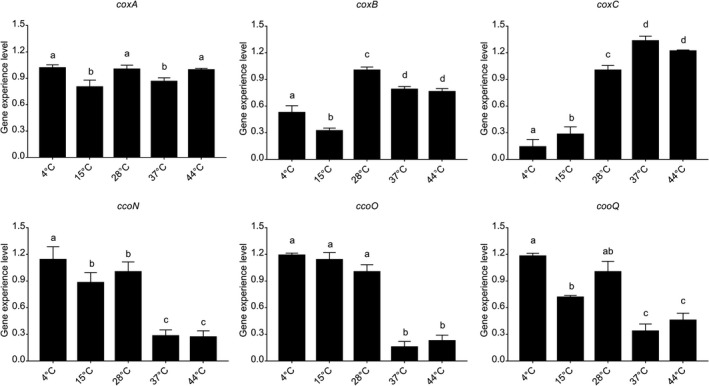
Temperature influenced the *Vibrio alginolyticus *adhesion*. *Quantitative real‐time PCR validation of the expression level of *coxA*, *coxB*, *coxC*, *ccoN*, *ccoO*, and *ccoQ *under different temperatures. Data are presented as the means ± *SD*, *n* = 6. Means of the treatments not sharing a common letter are significantly different at *p* < 0.05

Temperature affected the expression of *coxA*, *coxB*, *coxC*, *ccoN*, *ccoO*, and *ccoQ* in different ways (Figure 9). The expression levels of *ccoN*, *ccoO*, and *ccoQ* had no significant declined at 4, 15, and 28°C, while *ccoN*, *ccoO*, and *ccoQ* had their lowest expression levels at 37 and 44°C. The results showed that these three genes were more sensitive to higher temperatures. However, the expression trend of *coxC* was opposite to the trend of *ccoN*, *ccoO*, and *ccoQ*. *coxC* had the highest expression levels at 37 and 44°C, but had the lowest expression at 4 and 15°C. Interestingly, the expression of *coxA* had no significant difference at 4, 28, and 44°C, but the expression of *coxA* was significantly declined at 15 and 37°C. Whereas, the expression trend of *coxB* was different from that of the other genes. The *coxB* gene had the highest expression at 28°C.

The influence of temperature on *V. alginolyticus* adhesion was reported previously (Huang, Huang et al., 2016). The adhesion ability of *V. alginolyticus *under different temperatures displayed an inverted U‐shaped trend. The adherent number of *V. alginolyticus* to the fish mucus at 28°C was significantly higher than those at the other temperatures.

The bacterial adhesion assay and qRT‐PCR indicated that only the expression trend of *coxB* matched the results of bacterial adhesion assay. These results revealed that temperatures could affect the *V. alginolyticus* adhesion, but only the *coxB* gene probably take park in the adhesion regulation in response to temperature stimulus.

### 3.6 Influence of various pH treatments

**Figure 10:**
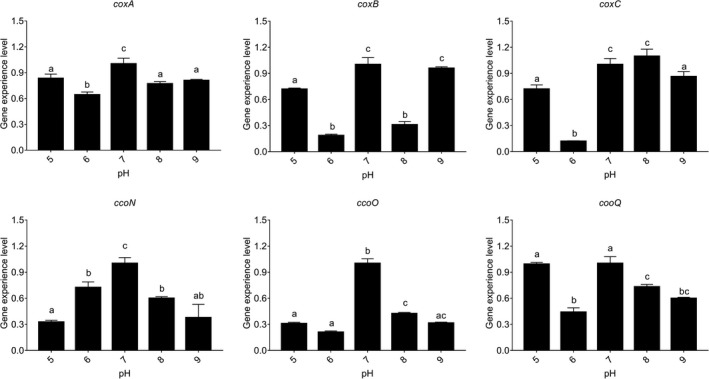
pH influenced the *Vibrio alginolyticus* adhesion. Quantitative real‐time PCR validation of the expression of *coxA*, *coxB*, *coxC*, *ccoN*, *ccoO*, and *ccoQ *under different pH. Data are presented as the means ± , *n* = 6. Means of the treatments not sharing a common letter are significantly different at *p* < 0.05

pH conditions significantly affected the expression of genes (Figure 10a). *coxA, coxB, ccoN, ccoO*, and *ccoQ *had the highest expression levels at pH 7.0, while *coxC* gene had the highest expression level at pH 8.0. But comparing with the expression level at pH 7.0, the expression level of *coxC* gene had no significant differences at pH 8.0. The results in Figure 10 also showed that all of these genes appeared to be sensitive to different pH except for *coxA *gene.

The *V. alginolyticus* adhesion ability to skin mucus at different pH values was investigated previously (Huang, Huang et al., 2016).The adhesion ability of *V. alginolyticus* reached its peak at pH 7.0, and the change of adhesion ability under different pH is also played an inverted U‐shaped trend. The adhesion of *V. alginolyticus* was decreased seriously with peracid and superalkalinity. This result is similar to Varma, Dinesh, and Menon (2010).

The results of in vitro adhesion assays and qRT‐PCR under different pH were quite similar except for *coxC* gene, while *ccoN *and *ccoO *had the highest similarity. These results indicated that pH could affect *V. alginolyticus* adhesion and the oxidative phosphorylation pathway probably be take part in the regulatory network governing adhesion under different pH, while *ccoN *and *ccoO *genes seemed to be more sensitive to different pH conditions.

### 3.7 Influence of different salinities

**Figure 11:**
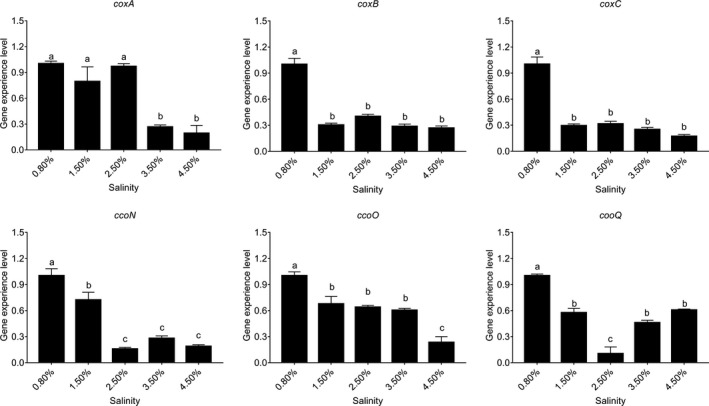
Salinity influenced the *Vibrio alginolyticus* adhesion. Quantitative real‐time PCR analysis of the expression of *coxA*, *coxB*, *coxC*, *ccoN*, *ccoO*, and *ccoQ *under different salinities. Data are presented as the means ± *SD*, *n* = 6. Means of the treatments not sharing a common letter are significantly different at *p* < 0.05

The influences of salinities on gene expression were quite different (Figure 11). The *coxB, coxC, ccoN*, and *ccoO *had the highest expression levels at the 0.80% salinity, while the expression levels of *coxB, coxC, ccoN*, and *ccoO *were significantly decreased from 1.50% salinity. The expression of *coxA* was not significantly changed at the 0.80%, 1.50%, and 2.50% salinity, while its expression was remarkably reduced at 3.50% and 4.50% salinity. The expression level of *ccoQ* displayed an U‐shaped trend and reached the lowest expression level at the 2.50% salinity.

The maximum adhesion was achieved at the 0.80% salinity (Huang, Huang et al., 2016) and this results was consistent with previous studies (Yan et al., 2007). The trend of in vitro adhesion assays and qRT‐PCR were similar at different salinities except for *ccoQ* gene. These results indicated that salinity could influence *V. alginolyticus *adhesion while *ccoQ* gene probably be not involved in the regulatory network governing adhesion in response to different salinities.

### 3.8 Effects of starvation

**Figure 12:**
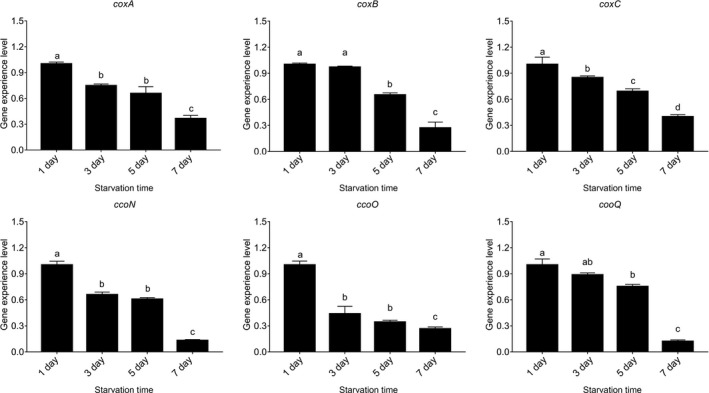
Starvation influenced the adhesion of *Vibrio alginolyticus.* Quantitative real‐time PCR analysis of the expression of *coxA*, *coxB*, *coxC*, *ccoN*, *ccoO*, and *ccoQ *after starvation for 1, 3, 5, and 7 days. Data are presented as the means ± *SD*, *n = *6. Means of the treatments not sharing a common letter are significantly different at *p* < 0.05

The results in Figure 12 showed that the expression levels of the six genes were significantly downregulated in a time‐dependent manner after starvation. According to our previous research (Huang, Huang et al., 2016), the viable bacteria number was not significantly changed at first 3 days of starvation, and the adhesion ability of *V. alginolyticus* was substantially reduced with the time prolonging. The decline of the number of *V. alginolyticus* adhered to skin mucus was mainly owe to the decline of the adhesion ability of *V. alginolyticus *rather than the declining number of bacteria in the suspension. These results were quite similar to the previous reports (Wang & Leung, 2000), which proved that vibriosis induced by *V. alginolyticus *was more likely to outbreak in eutrophic seawater rather than oligotrophic seawater (Huang, Huang et al., 2016). These results probably explain the decreased adhesion ability of starved *V. alginolyticus*.

Although the expression levels of *coxA, coxB, coxC, ccoN, ccoO*, and *ccoQ* of starved *V. alginolyticus* were impaired at 3 days, the expression levels of *coxB *and *ccoQ *had no significantly reduced. The starvation significantly downregulated the expression of *coxA *(by 1.34‐fold)*, coxC *(by 1.18‐fold)*, ccoN *(by 1.52‐fold)*, *and *ccoO *(by 2.29‐fold) at 3 days, respectively. Therefore, among these genes, *ccoO *appeared to be the more sensitive gene to starvation.

## 4 DISCUSSION

According to our previous reports, the adhesion of pathogens was a very complex process and was also a key step in the virulence of pathogens. In the previous researches of our laboratory, RNA‐seq was performed on *V. alginolyticus* which were treated with Cu^2+^, Pb^2+^, and low pH, and these stress conditions reduced the adhesion ability of *V. alginolyticus* significantly (Wang et al., 2015). Based on the RNA‐seq data and bioinformatics analysis, we found two pathways (including the TCA pathway and the flagellar assembly pathway) and three ncRNAs that might be related to adhesion of *V. alginolyticus*, which has been proved by qRT‐PCR, RNAi and in vitro adhesion assays (Huang, Xu et al., 2018). In the present study, the relationship between the oxidative phosphorylation pathway and adhesion ability of *V. alginolyticus* was investigated.

The results of our RNA‐seq analysis showed that there were six commonly downregulated DEGs in the oxidative phosphorylation pathway and the expression levels of these genes were significantly different in all stressed groups. Therefore, we hypothesized that these genes (including *coxA*, *coxB*, *coxC*, *ccoN*, *ccoO*, and *ccoQ*) might be sensitive to environmental stresses. Previous studies have shown that starvation (Monternier et al., 2015), pH (Fletcher, Feizi, Kim, Siewers, & Nielsen, 2015), temperature (Hu et al., 2015), and salinity are the important factors influencing distribution, abundance and diversity of aquatic animals (Xu et al., 2015). As Monternier et al. (2015) reported, starvation could decrease the activity of oxidative phosphorylation in skeletal muscle. In *Pichia anomala* cells, the oxidative phosphorylation were highly expressed when it grew in low pH (Fletcher et al., 2015). Seawater‐acclimated milkfish upregulated oxidative phosphorylation to produce more energy budget when they were under hypothermal stress (Hu et al., 2015). Tine, McKenzie, Bonhomme, and Durand (2011) also described that many genes of oxidative phosphorylation were relatively overexpressed in gill tissue with high salinity. But these studies only showed that the oxidative phosphorylation is sensitive to environmental stress, and have not shown the expression levels of genes involved in the oxidative phosphorylation pathway under the environmental stress. Hence, our present study could complement the defects, while the previous studies also indirectly supported our hypothesis.

In order to examine the relationship between *coxA*, *coxB*, *coxC*, *ccoN*, *ccoO*, and *ccoQ *and adhesion, qRT‐PCR, RNAi, bacterial adhesion assays, and CoxC activity assays were performed in this study. After the transient gene silencing (Figure 5), the expression levels of these genes were significantly impaired and the adhesion ability of *V. alginolyticus* was also significantly declined at the same time. These results indicated that *coxA*, *coxB*, *coxC*, *ccoN*, *ccoO*, and *ccoQ *were closely related to adhesion, which supported the results of RNA‐seq and our hypothesis. After the stable gene silencing, the results in Figure 6 showed that *coxC *gene had the lowest expression level among the six genes and the expression level of *coxA *gene was higher compared with *coxC*. Meanwhile, the expression level of *ccoQ *gene was the highest among these six genes. But the results in Figure 7 and Figure 8 showed that the adhesion ability and the activity of CoxC in *V. alginolyticus* had the most impaired after stable silencing of *ccoQ *gene, while *coxA *gene was on the heels. After stable silencing of *coxC *gene, the adhesion ability of *V. alginolyticus* and the activity of CoxC in *V. alginolyticus* had the least impaired. The previous reports by Iwata, Ostermeier, Ludwig, and Michel (1995) and Ekici, Pawlik, Lohmeyer, Koch, and Daldal (2012) supported our results.

As Iwata et al. described, the coxA is the biggest of the three subunits, and it is also a more important subunit. The CoxA is associated with CoxB at one side and CoxC at the other side. The coxA contains three redox centers: heme *a*, heme *a_3_*, and copper B (Cu_B_) (Iwata et al., 1995). Heme *a *is a low‐spin heme with two axial histidine ligands, while heme *a_3_* is a high‐spin heme with one histidine ligand (Michel, Iwata, & Ostermeier, 1999). The fast electron transfer between heme *a* and the binuclear heme *a_3_*_− _Cu_B_ redox centers was recently used to explain the high operational oxygen affinity of the oxidase by kinetic trapping of bound oxygen (Ostermeier, Iwata, & Michel, 1996). The heme *a_3_*_−_ Cu_B_ center is the catalytic core for oxygen reduction (Ostermeier et al., 1996). Obviously, the CoxA protein subunit plays an important role in electron translation and proton‐coupled electron transfer (Liu et al., 2014).

The CoxB is the subunit II of COXs which containing the binuclear Cu_A _center to receive the electrons from cytochrome C and transfer them to heme *a *and finally to the binuclear heme *a_3_*_−_ Cu_B_ center (Ostermeier et al., 1996).

As previously described, subunit III of CoxC contains no metal centers, so it has no direct function in electron transfer (Hosler, 2004). The pH and the proton affinity can influence the activity of CoxC. The CoxC has the ability to maintain proton flow to the active site, and it also exerts some structural influence that more directly protects the active site from destructive chemistry during the oxygen reduction cycle (Hosler, 2004). The CoxC connects with CoxA could contribute to optimum proton pumping efficiency (Hosler, 2004; Ostermeier et al., 1996).

Cytochrome C oxidase, a protein complex located in the inner membrane of mitochondria in eukaryotic and many prokaryotic aerobic organisms (Soulimane et al., 2000), is commonly abbreviated to COX (Ostermeier et al., 1996), CcO (Naruta et al., 2001), CytcO (Johansson et al., 2011), and complex IV (Barrientos, Gouget, Horn, Soto, & Fontanesi, 2009). The enzyme is the terminal enzyme of most respiratory chains (Iwata et al., 1995), which catalyzes the reduction of molecular oxygen to water concomitant with the oxidation of reduced cytochrome C and uses the free energy derived from this reaction to pump protons across a membrane (Johansson et al., 2011). Eukaryotic COX is consisted of 11–13 subunits (11 in the yeast *Saccharomyces cerevisiae* and 13 in mammals) (Barrientos et al., 2009). Prokaryotic COX usually have a less subunits, four subunits as usual (Iwata et al., 1995). However, subunits I (CoxA), II (CoxB), and III (CoxC) of CoxC are highly degree of sequence conserved from bacteria to humans (Hosler, 2004). These three subunits are essential for all of the aa_3_‐type bacterial CoxCs (Hosler, 2004). Although there has been no reports on the relationship between *coxA*, *coxB, coxC *and bacterial adhesion or virulence, it has been reported that when the activity of mitochondrial CoxC is reduced and the cell adhesion ability also declined (Karu & Pyatibrat, 2011).

As Lohmeyer et al. (2012) described that the *cbb_3_*‐Cox could replace the function of *aa_3_*‐type cytochrome oxidases when the bacteria lack *aa_3_*‐type cytochrome oxidases. The *cbb_3_*‐type cytochrome oxidase (*cbb_3_*‐Cox), without a Cu_A_ center present, is a proton‐pumping terminal oxidase solely in bacteria (Trasnea et al., 2018), and it is crucial for anoxyenic photosynthesis and nitrogen fixation. In addition, the *cbb_3_*‐type cytochrome oxidase is important for many pathogenic bacteria to colonize under the low oxygen tissues. The enzyme has a high apparent affinity for oxygen, and it allows these pathogens to colonize low oxygen containing tissues (Ekici et al., 2012). Reducing molecular oxygen at low oxygen concentration, the enzyme is not based on a particularly fast binding of O_2_, but rather on the irreversibility of the O_2_ binding to it (Rauhamäki, Verkhovsky, & Wikström, 2008). The previous studies have shown that the activity of *cbb_3_*‐Cox is dependent on the environmental oxygen concentrations (Ekici et al., 2012). The activity is high under microaerobic, low under fully aerobic, and even lower under fully anaerobic growth condition (Ekici et al., 2012). The *cbb_3_*‐Cox is consisted of CcoN (subunit I), CcoO (subunit II), CcoP (subunit III), and CcoQ (subunit IV) proteins (Ekici et al., 2012; Rauhamäki et al., 2008). CcoO is *c*‐type cytochromes with a monoheme. It conveys electrons to heme *b* of CcoN and makes strong contacts with the alpha‐helices of CcoN (Ekici et al., 2012). CcoP is also a C‐type cytochromes with a diheme (Ekici, Jiang, Koch, & Daldal, 2013). It conveys electrons to the heme group of CcoO (Ekici et al., 2012). Meanwhile, the main catalytic subunit of *cbb_3_*‐Cox is CcoN, which is a conserved 12 transmembrane helices containing membrane protein with a binuclear center heme *b_3_*‐Cu (Ekici et al., 2013). CcoQ is a small subunit formed by a single transmembrane helix, and is not present in all *cbb_3_*‐type cytochrome oxidase (Ekici et al., 2012). It does not contain any cofactor, and elimination does not completely abolish *cbb_3_*‐type cytochrome oxidase activity (Ekici et al., 2012), but it is thought to enhance the stability of the *cbb_3_*‐Cox (Ekici et al., 2013). From the results of stable gene silencing, when the expression level of *ccoQ* gene was reduced to a lesser extent, the adhesion ability and the activity of CoxC in *ccoQ*‐RNAi *V. alginolyticus *were greatly decreased. Thus, the subunit CcoQ was not only enhanced the stability of the *cbb_3_*‐Cox, but also played an important role in the adhesion ability and the pathogenicity of *V. alginolyticus*. The result of this study complements this deficiency.

Not only on eucaryon, there is now abundant literature on the three different types of CoxCs and oxidative phosphorylation that are encountered in prokaryotes (such as *Rhodobacter sphaeroides, Bacillus alcalophilus, *cyanobacterium* Anabaena variabilis*, *E. coli*, and *Pseudomonas stutzeri*) (Cukier, 2005; Galván et al., 2018; Guffanti, Bornstein, & Krulwich, 1981; Hempfling & Hertzberg, 1979; Kohlstaedt, Buschmann, Langer, Xie, & Michel, 2017; Kohlstaedt et al., 2016; Melin et al., 2018, 2016; Schmetterer et al., 2001; Sun, Benlekbir et al., 2018; Sun, Luo et al., 2018). At the same time, the *cbb_3_*‐Cox is only in bacteria and is the sole Cox in pathogenic bacteria. Up to now, some studies have validated the function of the *cbb_3_*‐Cox in some pathogenic bacteria (such as *Campylobacter jejuni*, *Helicobacter pylori*, *Neisseria meningitides*, and *Neisseria gonorrhoeae*) (Ekici et al., 2012), whereas there is few studies about CoxC, *cbb_3_*‐type cytochrome oxidase, and oxidative phosphorylation pathway in *V. alginolyticus*. Therefore, our present study not only investigated CoxC, *cbb_3_*‐type cytochrome oxidase, and oxidative phosphorylation pathway in *V. alginolyticus, *but also verified the relationship between the oxidative phosphorylation pathway and *V. alginolyticus* adhesion at the molecular level and indicated how the environmental factors influenced *V. alginolyticus* adhesion through oxidative phosphorylation pathway.

In this study, with RNAi, qRT‐PCR, and adhesion assays, we investigated the relationships among the six genes, adhesion ability and environmental stresses (including temperature, pH, salinity, and starvation). The results showed that the *V. alginolyticus* adhesion ability was substantially affected by environmental factors. The six genes in the oxidative phosphorylation pathway showed different responses to different environmental stresses. Whereas, the same environmental stress leads to different changes in expression of different genes. These results indicated that the oxidative phosphorylation pathway played an important role in the *V. alginolyticus* adhesion and was sensitive to some environmental stresses, which was consistent with previous reports (Huang, Huang et al., 2016).

In summary, the present study demonstrated that the oxidative phosphorylation pathway was sensitive to environmental factors, and it was related to the adherence of *V. alginolyticus.*

## CONFLICT OF INTEREST

The authors declared that they have no conflicts of interest to this work.

## AUTHORS CONTRIBUTION

Q. Y. and Y. S. conceived and designed the experiments. L‐X. H., L. H., L. Z., and Y. Q. performed the experiments. Q. Y. and L‐X. H. drafted and revised the manuscript. All the authors approved the final version.

## ETHICS STATEMENT

All animal experiments were carried out strictly under the recommendations in the “Guide for the Care and Use of Laboratory Animals” set by the National Institutes of Health. The animal protocols were approved by the Animal Ethics Committee of Jimei University (Acceptance No. JMULAC201159).

## Data Availability

The data of RNA‐seq was deposited in the NCBI SRA (accession number: SRP049226).
